# Effects of the winter temperature regime on survival, body mass loss and post-winter starvation resistance in laboratory-reared and field-collected ladybirds

**DOI:** 10.1038/s41598-020-61820-7

**Published:** 2020-03-18

**Authors:** Michal Knapp, Michal Řeřicha

**Affiliations:** Department of Ecology, Faculty of Environmental Sciences, Czech University of Life Sciences Prague, Kamýcká 129, Prague, Suchdol 165 00 Czech Republic

**Keywords:** Invasive species, Ecophysiology

## Abstract

Ongoing climate change results in increasing temperatures throughout the seasons. The effects of climate change on insect performance are less studied during the winter season than during the growing season. Here, we investigated the effects of various winter temperature regimes (warm, normal and cold) on the winter performance of the invasive ladybird *Harmonia axyridis* (Coleoptera: Coccinellidae). Winter survival, body mass loss and post-winter starvation resistance were measured for a laboratory-reared population as well as three populations collected from the field prior to overwintering. The warm winter regime increased the survival rate and body mass loss and reduced post-winter starvation resistance compared to those of the ladybirds in the cold winter regime. The effects of the temperature regime were qualitatively similar for the laboratory-reared and field-collected beetles; however, there were significant quantitative differences in all measured overwintering parameters between the laboratory-reared and field-collected populations. The winter survival of the laboratory-reared beetles was much lower than that of the field-collected beetles. The laboratory-reared beetles also lost a larger proportion of their body mass and had reduced post-winter starvation resistance. Winter survival was similar between the females and males, but compared to the males, the females lost a smaller proportion of their body mass and had better post-winter starvation resistance. The pre-overwintering body mass positively affected winter survival and post-winter starvation resistance in both the laboratory-reared and field-collected ladybirds. The significant differences between the laboratory-reared and field-collected individuals indicate that quantitative conclusions derived from studies investigating solely laboratory-reared individuals cannot be directly extrapolated to field situations.

## Introduction

An increasing body of literature shows that temperature is one of the most important factors determining the distribution of organisms on the globe^1–3^. Also the distribution of insect species is strongly affected by temperature, and this relationship is especially apparent for winter temperatures^4,5^. Overwintering insects have to overcome stressful environmental conditions, e.g., unavailable food resources, low temperatures or water deficits, and thus, it is unsurprising that many insect species suffer from substantial mortality during the winter period^5^. In response, insects have adopted complex strategies to overcome stressful winter conditions^6,7^.

The low temperatures experienced during overwintering can result in the mortality of insects due to severe tissue damage caused by ice crystallization within cells or due to accumulated chill injuries resulting in metabolic disruptions, even at non-freezing temperatures^7^. In general, insects may adopt one of two strategies to mitigate the danger of internal ice formation: either tolerate freezing (i.e., withstand the formation of ice) or avoid freezing (i.e., reduce the freezing point to a very low temperature^8^). The temperature of water crystallization is close to 0 °C, and ice is usually restricted to the extracellular compartments in freeze-tolerant species. Osmotic dehydration ensures that water does not freeze inside cells^9^. In freeze-avoiding species, removing ice nucleators and accumulating antifreezing substances results in liquid bodily fluids at temperatures well below the melting point of water^10–12^. However, the majority of insects, including the harlequin ladybird *Harmonia axyridis* (Coleoptera: Coccinellidae) investigated in this study, are chill-susceptible species, for which even low temperatures well above the melting point cause chill injuries and can induce a reversible state of immobility called “chill coma”^13,14^.

As food resources are commonly very limited during the winter period, especially in temperate zones, the management of energy reserves is crucial for insects to successfully overwinter. A great majority of insect species overwinter in a dormant state (quiescence or diapause) in which metabolic rates are suppressed^6,15^, but they are still temperature dependent^5^. Quiescence is a reversible state of very low activity with suppressed metabolism, but insects remain highly responsive to environmental cues, and their activity, e.g., feeding or reproduction, can be renewed quickly^16^. In contrast, diapause is a hormonally determined state, including the complex “diapause syndrome”, i.e., the modification of insect physiology (e.g., the investment in body fat growth and slowdown of ovariole development) as well as behaviour (e.g., the selection of protected microhabitats^6,15,17^). Some insects are able to evaluate their energy reserves during diapause and to terminate diapause prematurely. However, the termination of diapause is a gradual process that can take several weeks; thus, insects exposed to suboptimal conditions during diapause can be significantly negatively affected^15^. In some species, high winter temperatures linked to a relatively high energy drain can result in enhanced winter mortality, reduced spring longevity or limited fecundity during the following growing season^18,19^.

Insects have a limited ability to regulate their body temperature, and thus, ongoing climate change is expected to have serious fitness consequences^5,20^. However, the majority of climate change research is focused on the effects taking place during the growing season, whereas far less is known about the effects of the increased temperatures experienced by insects during the winter period^4,5^. The link between the winter air temperatures and the temperatures experienced by overwintering insects is not always clear. As an example, reduced snow cover can result in relatively low winter temperatures for insects overwintering in the upper soil layer^4^. Moreover, species-specific responses to temperature preclude any generalization of the effects of relatively high winter temperatures across species. The positive effects of increased winter temperatures have been reported, e.g., for *Drosophila suzukii*, *Nezara viridula* and *Halyomorpha halys*^20–22^, but negative effects are as common, e.g., for *Erebia medusa*, *Osmia lignaria* and *Chilo suppressalis*^23–25^. As climatic changes seem to be linked to shifts in species distribution^26,27^, knowledge of the effects of relatively high winter temperatures can be especially important for predicting potential distribution changes in invasive species.

In general, the investigation of the effects of climate change on overwintering insects is bound to several potential methodological problems. The temperature treatment should be set to realistically mimic ongoing climate change: a very large temperature increase will surely induce a significant response in the experimental insects but is improbable in nature. The commonly employed practice of exposing insects to unrealistic constant temperatures would not provide reliable results, as temperature fluctuations naturally occur in the field^28,29^. Not mean but extreme temperatures, i.e., daily minima or maxima, can drive the observed effects of temperature on insects^30^. In many cases, laboratory-reared insects are employed in overwintering studies, and insects that experienced unnatural environmental conditions prior to overwintering may respond differently than wild insects to the elevated overwintering temperatures (e.g., lower winter survival in the laboratory-reared *H. axyridis* individuals compared to the field-collected ones^31^). In this study, we did our best to address all these methodological challenges.

We aimed to investigate the effects of warm, normal and cold winter temperatures, based on real long-term meteorological data, on overwintering survival, body mass loss during the winter and post-winter starvation resistance in the invasive ladybird *H. axyridis*. *Harmonia axyridis* is an interesting model species as it has been considered to be one of the most invasive insect species in Europe and North America^32,33^. *Harmonia axyridis* is native to Asia, particularly to areas with a temperate and subtropical climate^34^, but has recently spread to many European countries, North and South America and some African countries^35–37^. The speed of their range expansion in a novel environment has been extremely fast because in 15 years (2000–2015), the species spread over almost the whole of Europe^34,38^. However, its spread seems to be partially limited by the environmental conditions, especially high summer temperatures, as its thermal optimum is lower than that of many other ladybird species^39^. In general, in Europe, *H. axyridis* has not spread into relatively high altitudes (above ca. 2000 m a.s.l.), indicating a possible problem with overwintering there^35^. However, note that during the summer season, *H. axyridis* adults were recorded at much relatively high altitudes (ca. 3500 m a.s.l.) in the Andes^40^.

*Harmonia axyridis* overwinters as an adult, which is very common in beetles, especially in ladybirds^41,42^. In the autumn, during warm and sunny days, mass flights to overwintering sites can be observed^35^. *Harmonia axyridis* commonly overwinters in aggregations (from tens to several thousands of individuals) and prefers indoor sites or shelters that buffer the low winter temperatures (e.g., window frames, old buildings or caves^43^). The adults have only a weak winter diapause, commonly terminated in January and followed by a quiescent state^44^. The total time spent in diapause can be variable. Such flexibility in diapause behaviour may be an important factor that contributes to the invasive success of *H. axyridis*^44,45^. In the spring, commonly from March to April, the beetles disperse slowly from their hibernation sites (after quiescence termination) towards their feeding and breeding sites^35,42^. It is a common phenomenon that ladybirds suffer from starvation during the early spring as their preferred food resources (aphids) are rare at that time^42^.

We hypothesize that higher winter temperatures will result in increased survival rates due to decreased chill injuries^14^, but at the cost of reduced post-overwintering conditions, i.e., a greater body mass reduction during the winter and reduced post-overwintering survival due to the higher energy consumption at higher winter temperatures^19^. We also compared the results obtained for the laboratory-reared and field-collected populations to reveal the potential biases linked to the use of laboratory insects in ecophysiological studies and investigated the effects of sex and pre-winter body mass on overwintering survival, body mass loss during winter and post-winter starvation resistance in *H. axyridis*.

## Results

### Effects of the temperature regime

**Figure 1:**
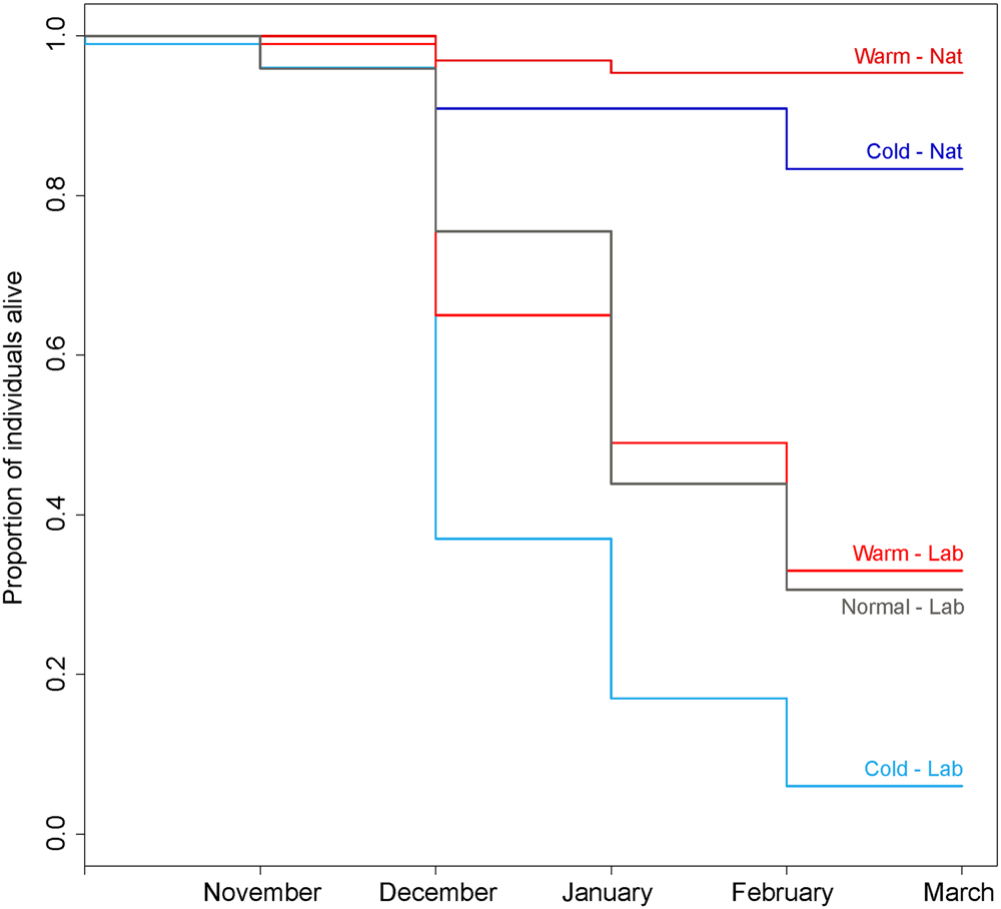
Winter survival of adult *Harmonia axyridis* ladybirds under different temperature regimes. Cold, normal and warm winter conditions are represented by the blue, grey and red colours, respectively. Laboratory-reared (Lab) and field-collected (Nat) beetles are represented separately, as there were significant differences in their survival probabilities. The data for the females and males were pooled, as there were no significant differences in their survival. For the same reason, we also pooled the data from all three different natural populations.

**Figure 2:**
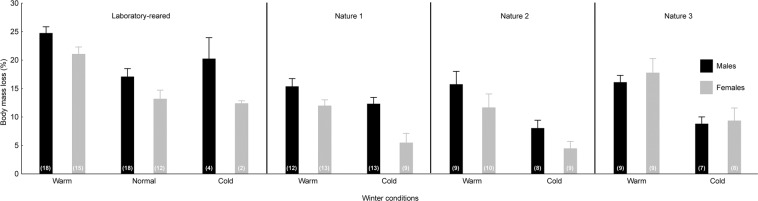
Relative body mass loss of *Harmonia axyridis* during overwintering under the different temperature regimes. Laboratory-reared and field-collected beetles (Nature 1–3) are represented separately. The females (grey columns) and males (black columns) are shown separately. The mean values + SEMs are shown. The numbers in the brackets represent the sample sizes.

**Figure 3:**
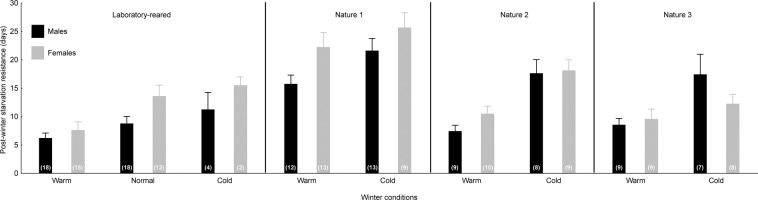
Post-winter starvation resistance of *Harmonia axyridis* after overwintering under the different temperature regimes. Laboratory-reared and field-collected beetles (Nature 1–3) are represented separately. The female (grey columns) and male (black columns) post-winter starvation resistance (longevity without food in days) is shown separately. The mean values + SEMs are shown. The numbers in the brackets represent the sample sizes.

We found qualitatively consistent effects of the winter temperature on survival, body mass loss and post-winter starvation resistance across all the investigated populations (one laboratory-reared and three field-collected; Tables 1 and 2). Exposure to low winter temperatures (cold regime) resulted in a significantly reduced winter survival (Fig. 1), a reduced body mass loss (Fig. 2) and improved post-winter starvation resistance (Fig. 3) compared to those in the exposure to high winter temperatures (warm regime). For detailed comparisons of the differences among all three temperature regimes for the laboratory-reared animals, see the post hoc test values in Table 1.

**Table 1:** Effects of temperature regime and sex on survival, relative body mass loss and post-winter starvation resistance in laboratory-reared *Harmonia axyridis*.

Term	Survival		Relative body mass loss		Post-winter starvation resistance	
	Χ^2^-value	P-value	F-value	P-value	F-value	P-value
Temperature regime	61.48	**<0.001**	17.47	**<0.001**	6.31	**0.004**
Sex	0.00	0.987	11.26	**0.002**	8.13	**0.006**
Pre-overwintering body mass	37.49	**<0.001**	3.80	0.057	6.58	**0.013**
Temp × Sex	0.20	0.904	0.41	0.665	0.06	0.940
Temp × Mass	2.94	0.230	0.75	0.480	0.64	0.530
Sex × Mass	7.99	**0.005**	0.10	0.750	0.13	0.718
Temp × Sex × Mass	3.00	0.223	0.24	0.790	0.62	0.543
Treatment contrasts for the temperature regimes (Tukey’s HSD)	Warm	b	Warm	b	Warm	a
	Normal	b	Normal	a	Normal	b
	Cold	a	Cold	ab	Cold	b

Parental pair was included as a random effect in all models. All significant terms are highlighted in bold. The results of the Tukey’s HSD post hoc tests are shown for comparisons between particular temperature regimes (unique letters indicate significant differences between the temperature regimes).

**Table 2:** Effects of temperature regime, sex and population identity on survival, relative body mass loss and post-winter starvation resistance in *Harmonia axyridis*.

Term	Survival		Relative body mass loss		Post-winter starvation resistance	
	Χ^2^-value	P-value	F-value	P-value	F-value	P-value
Temperature regime	16.54	**<0.001**	93.01	**<0.001**	66.96	**<0.001**
Sex	0.07	0.794	20.52	**<0.001**	5.92	**0.016**
Population identity	187.69	**<0.001**	25.52	**<0.001**	33.76	**<0.001**
Pre-overwintering body mass	25.85	**<0.001**	0.69	0.407	23.38	**<0.001**
Temp × Sex	0.01	0.927	0.86	0.356	3.39	0.068
Temp × Pop	0.68	0.877	0.70	0.556	2.45	0.066
Sex × Pop	4.18	0.243	2.64	0.052	1.13	0.341
Temp × Mass	1.36	0.243	0.05	0.826	0.15	0.701
Sex × Mass	7.22	**0.007**	0.14	0.712	4.18	**0.043**
Pop × Mass	3.72	0.293	2.05	0.111	0.77	0.511
Temp × Sex × Pop	9.27	**0.026**	0.30	0.823	0.58	0.630
Temp × Sex × Mass	2.15	0.143	1.07	0.304	0.58	0.449
Temp × Pop × Mass	1.49	0.683	1.00	0.397	1.14	0.335
Sex × Pop × Mass	0.91	0.822	0.52	0.668	1.14	0.337
Temp × Sex × Pop × Mass	0.00	1.000	0.33	0.807	0.51	0.679
Treatment contrasts for population identity (Tukey’s HSD)	Laboratory	a	Laboratory	b	Laboratory	a
	Nature 1	b	Nature 1	a	Nature 1	c
	Nature 2	b	Nature 2	a	Nature 2	b
	Nature 3	b	Nature 3	a	Nature 3	ab

The analysed temperature regimes are limited only to ‘cold’ and ‘warm,’ as data for the ‘normal’ temperature regime are not available for the field-collected populations. All the significant terms are highlighted in bold. The results of the Tukey’s HSD post hoc tests are shown for comparisons between particular investigated populations (unique letters indicate significant differences between populations).

### Differences between the laboratory-reared and field-collected populations

The qualitatively similar effects of the winter temperature regime on winter survival, body mass loss, and post-winter starvation resistance were observed across the investigated populations, but the absolute values differed significantly between the populations (mainly, the laboratory-reared beetles differed from all the field-collected populations). The laboratory-reared beetles had significantly lower winter survival compared to that of the field-collected ladybirds (Fig. 1; Table 2). Additionally, the laboratory-reared beetles suffered a significantly higher body mass loss during overwintering compared to that of the field-collected ladybirds (Fig. 2; Table 2). Two out of three field-collected ladybird populations (‘Nature 1’ and ‘Nature 2’) outperformed the laboratory-reared beetles in post-winter starvation resistance (Fig. 3; Table 2).

### Differences between the sexes

There were no significant differences in winter survival between the males and females (Tables 1 and 2). However, there were significant differences between the sexes in relative body mass loss during winter and in post-winter starvation resistance (Tables 1 and 2). The females lost a smaller proportion of their body mass during overwintering compared to that of the males (Fig. 2). Note that also the absolute body mass loss differed between the sexes, while body mass was reduced more in males compared to females (P < 0.001). Apart from the ‘Nature 3’ population, the females outperformed the males in post-winter starvation resistance (Fig. 3).

### Effects of pre-overwintering body mass

Pre-overwintering body mass significantly affected winter survival in *H. axyridis*. The relatively heavy individuals, both laboratory-reared and field-collected, had increased survival probabilities, and this effect was stronger for the males compared to the females (see the significant interaction between sex and pre-overwintering mass in Tables 1 and 2; Supplementary Material Fig. S1). Pre-overwintering mass had no effect on body mass loss during the winter, indicating that individuals with a high pre-winter body mass lost a proportion of their live mass similar to that of the individuals with a low pre-winter mass (Tables 1 and 2). High pre-overwintering mass had a significant positive effect on post-winter starvation resistance, and this effect was stronger for females compared to males (see the significant interaction between sex and pre-overwintering mass in Tables 1 and 2). Interestingly, the significantly higher pre-overwintering mass achieved by the laboratory-reared beetles (Supplementary Material Fig. S2) was not able to ensure a winter performance comparable to that of the field-collected beetles (Figs. 1–3), and thus, the positive effect of pre-overwintering body mass is significant mainly at the intra-population level, i.e., when the performance of individuals originating from the same population is compared.

## Discussion

### Main findings

In the present study, we demonstrated contrasting effects of the winter temperature regime on the winter survival, body mass loss and post-winter starvation resistance of the invasive ladybird *H. axyridis*. Lower winter temperatures significantly decreased the survival probability, reduced body mass loss and enhanced post-winter starvation resistance compared to those of higher winter temperatures. Interestingly, there were also significant differences in the majority of the investigated parameters between the laboratory-reared and field-collected beetles, indicating that the results of the ecophysiological measurements performed solely on laboratory-reared animals need to be interpreted with caution.

### Effects of the temperature regime

Our temperature regimes did not represent very extreme winter conditions for *H. axyridis*, as they were far from the physiological limits of the species (the lower lethal temperature is approximately −16 °C in the European population of *H. axyridis*^31^). Applied temperatures are also completely within the range of the winter temperatures experienced by naturally overwintering *H. axyridis* adults in Central Europe (Řeřicha, unpublished temperature measurements at various overwintering sites). Despite this fact, the cold temperature regime (lower winter temperatures) caused significantly higher ladybird mortality than did the warm winter temperature regime. A possible explanation could be the long-term accumulation of chill injuries that can take place even at temperatures much higher than the lower lethal temperature (measured in short-term laboratory assay^4^). Energy exhaustion is unlikely to be the cause of death in our overwintering beetles, as the energy reserves (body mass) of the surviving beetles were higher in the cold regime than in the warm regime. In general, the energy consumption in overwintering insects increases with temperature within the range of ecologically relevant temperatures. This pattern is driven by the temperature dependence of metabolic rates^5^. This pattern was also confirmed for *H. axyridis* by our post-winter starvation resistance experiment. The ladybirds exposed to the cold regime survived under starvation conditions for a longer time than the individuals exposed to the warm regime.

Such a complex effect of the winter temperature regime complicates predictions of the effects of climate change on overwintering insects. With ongoing climate change, many insect species benefit from increased minimum winter temperatures during the coldest months in terms of reduced mortality due to chill injuries^21,46^. On the other hand, a substantial proportion of species suffer from enhanced energy demands due to higher autumn and early spring temperatures^47^. The relative importance of chill injury reduction and increased energy exhaustion might be species-specific, as species differ substantially in their traits, such as cold tolerance, energy acquisition abilities, diapause duration, and energy demands in the spring^5,47^. Research on different insect species have shown that the effects of climate change on insect overwintering success range from highly positive to highly negative. For example, higher winter temperatures increased survival of *Nezara viridula*, *Drosophila suzukii* and *Halyomorpha halys*^20–22^, but reduced survival of *Osmia lignaria*, *Erebia medusa* and *Chilo suppressalis*^23–25^. To reveal the overall effects of winter temperature, more complex experiments tracking insects throughout the complete life cycle are needed^20^, as post-overwintering performance, e.g., reproductive success, is crucial^5^. Based on the published literature, it is unfeasible to predict whether increased temperatures during overwintering will result in enhanced fitness in *H. axyridis*. Therefore, future studies investigating the performance of ladybirds that have experienced various winter temperature regimes during the following spring and summer seasons are needed. Moreover, ongoing climate change could result in an increased variability of environmental conditions during winter in many regions. Repeated switching between detrimental high and low temperatures can be exceptionally challenging for many insect species^4^.

### Differences between the laboratory-reared and field-collected populations

Differences observed in the overwintering performance of the three field-collected *H. axyridis* populations can be caused by different genetic background or environmental conditions experienced in nature, i.e., phenotypic differences, both representing a common source of intraspecific variation in insects^4,21,25,48^. Lower post-winter starvation resistance recorded by survival Population originating from České Budějovice could be linked to exposition to elevated temperatures during autumn transportation to Prague. However, we have no simple explanation for the difference between populations Nature 1 and Nature 2, both originating from Prague. Much more pronounced differences were observed between the field-collected and laboratory-reared individuals for all measured parameters. Even larger differences in *H. axyridis* winter survival between the laboratory-reared and field-collected individuals were observed by Berkvens *et al*.^31^; however, their laboratory beetles probably did not enter diapause at all. Our experimental ladybirds seemed to be diapausing, as exposure to room temperature did not result in higher movement activity in November and December. Despite our effort to mimic autumnal conditions in the laboratory, some natural cues remain difficult to mimic under laboratory conditions. For example, continuous changes in food quality and quantity during autumn or a long flight to an overwintering site may have resulted in better physiological readiness for overwintering among the field-collected ladybirds. The differences in insect performance between the laboratory-reared and field-collected individuals are not limited only to ladybirds and overwintering conditions^22,49^. Thus, we recommend conducting physiological and fitness measurements on both populations simultaneously if possible, as the laboratory populations could not provide reliable estimates of the field performance. It should also be noted that our laboratory beetles were relatively young (up to 30 days old) when the overwintering experiment started, whereas the field-collected beetles were of unknown age but probably much older on average (adults of the relatively large ladybird species can live for more than one year under field conditions^42^). Differences in ladybird age can significantly affect their winter performance, as physiological traits, including those related to overwintering, vary significantly, especially across the life of young adults^13,50^.

### Differences between the sexes and effects of pre-overwintering body mass

In general, females are the larger sex in *H. axyridis*^51^, and this was also true for the beetles investigated in this study. Our body mass loss data revealed that both the absolute and the relative body mass loss were higher in males compared to females. Thus, a smaller relative body mass loss in females cannot be linked solely to the existence of sexual size dimorphism, but a sex-specific management of energy reserves probably exists in *H. axyridis*. The larger female body mass may be responsible for the enhanced post-winter starvation resistance in the *H. axyridis* females, as a larger body size can be linked to the advantages of relatively larger energy stores and lower mass-specific metabolic rates^52^. However, based on our data, it is not possible to reject the possibility that *H. axyridis* females have also a sex-specific physiological adaptation enabling them to survive longer in the spring under starvation conditions, as was shown for the carabid beetle *Anchomenus dorsalis*^53^. On the other hand, our results clearly indicate that even within the sexes, a larger pre-overwintering body mass was linked to enhanced winter survival and post-winter starvation resistance. A greater pre-overwintering body mass can represent both individuals with a larger structural body size, e.g., a longer body length, and individuals in better body condition, e.g., higher energy reserves^54^. There is evidence that both structural size and body condition can affect starvation resistance^53,55^ as well as winter survival^56,57^ in insects. Without measurements of the structural body size in this study, we are not able to clearly distinguish whether heavier *H. axyridis* individuals were only larger, i.e., with a larger structural body size, or in better condition, i.e., with higher energy reserves.

## Conclusions

In conclusion, our results indicate that even small changes in winter temperatures can significantly affect winter mortality, winter body mass loss and post-winter starvation resistance in *H. axyridis*. While elevated temperature has contrasting effects on winter survival (positive) and post-overwintering energy reserves (negative), its overall effects on the population growth of *H. axyridis* remain unknown and should be investigated in a future study. The significant differences in overwintering performance between the laboratory-reared and field-collected individuals indicate that the quantitative conclusions derived from studies investigating solely laboratory-reared individuals cannot be directly extrapolated to field situations. We recommend employing both field-collected and laboratory-reared individuals in studies investigating physiological traits in insects if this approach is feasible.

## Materials and methods

### Experimental insects

Ladybirds originating from two sources were employed in our experiment: 1) laboratory-reared ladybirds and 2) field-collected ladybirds. The parents of our laboratory-reared ladybirds were collected in August 2015 from shrubs and lime trees on the university campus of the Czech University of Life Sciences Prague, Czech Republic (GPS: 50°8′N, 14°21′E; 300 m a.s.l.). After transportation to the laboratory, the ladybirds were sexed, and 20 parental pairs were formed at random (for details see^13^). Each couple was placed in a separate Petri dish (9 cm in diameter) containing crumpled filter paper strips, which provided a suitable substrate for egg laying. The Petri dishes were placed into a computer-controlled climatic chamber (made on order by the AVIKO-PRAHA company, Czech Republic) set to a 16 L:8 D photoperiod, 70% humidity and a temperature of 26 °C. From the reproducing pairs, 10 pairs were selected at random, and their offspring became our laboratory-reared beetles. Laboratory-reared beetles were also exposed to the standardized laboratory conditions (16 L:8 D photoperiod, 70% humidity, 26 °C) throughout their preimaginal development. The selected temperature is optimal for rearing *H. axyridis*^42,51^. The newly hatched larvae were fed ad libitum with the eggs of *Ephestia kuehniella* (Zeller, 1879) (Lepidoptera: Pyralidae) and provided water in cotton wool. The adult laboratory-reared ladybirds (emerged between September 15^th^ and 25^th^, 2015) were sexed, placed individually into Petri dishes, provided food and water ad libitum and exposed to the same standardized conditions as the larvae until October 7^th^, when the ladybirds were transferred to an 18 °C and 12 L:12 D photoperiod regime to initiate winter diapause. Thirty beetles (15 males + 15 females) from each parental pair were employed in our overwintering experiment, i.e., 300 laboratory-reared ladybirds in total.

In addition to the laboratory-reared ladybirds, adult ladybirds collected in the field in late autumn 2015 (mid-October) were employed in our overwintering experiment (field-collected beetles). We collected three geographically distinct *H. axyridis* populations when the beetles were aggregating at overwintering sites in Prague–Farkáň (Nature 1: 26 males + 26 females; aggregation on a building), Prague–Prokopské údolí (Nature 2: 20 males + 20 females; aggregation on pine trees) and České Budějovice (Nature 3: 19 males + 20 females; aggregation on a building). These field-collected ladybirds were then stored in groups in glass jars under natural outdoor conditions for 2–3 days until the beginning of the overwintering experiment. Ladybirds originating from České Budějovice also spent one additional day at higher temperature due to their transportation to Prague.

### Experimental setup and measurements

The overwintering experiment was started on October 15^th^, when all the ladybirds (both laboratory-reared and field-collected) were weighed for live mass (pre-overwintering mass) using a Sartorius balance with a precision of 10^−4^ g. At the same time, field-collected beetles were individually accommodated in Petri dishes, and all the ladybirds were transferred to computer-controlled climatic chambers set to a pre-overwintering regime with a low fluctuating temperature and a short photoperiod [8 L (12 °C):16 D (6 °C)] to mimic the outdoor conditions in late autumn. Six days later, the overwintering phase started, and the beetles were assigned to three winter temperature regimes: (1) warm winter, (2) normal winter and (3) cold winter. These temperature regimes mimicked the real temperatures experienced in outdoor shelters in Prague, i.e., the temperature 1 cm below the soil surface. Based on long-term meteorological data (November to March temperatures from 1995 to 2015) from the meteorological station situated in the Crop Research Institute, Praha-Ruzyně, extreme years with cold and warm temperatures were selected. The temperature course for our normal thermal regime was computed as the mean winter temperature course for the non-extreme years (from 1995 to 2015). The original meteorological data represented hourly mean temperatures (measured 1 cm below the soil surface). The data used as the normal thermal regime in our experiment thus represent the mean hourly temperature, averaged across multiple years. Three computer-controlled climatic chambers, one for each temperature regime (i.e., warm, normal or cold), were adjusted every hour with a new temperature value following the data in Supplementary Material File Table S1. The mean temperature (November to March) for the warm regime was 2.8 °C (the minimum temperature reached was −2.0 °C), the mean temperature for the normal regime was 0.7 °C (the minimum temperature reached was −4.1 °C) and the mean temperature for the cold regime was −1.6 °C (the minimum temperature reached was −8.1 °C). The laboratory-reared ladybirds were exposed to all three temperature regimes (five males and five females per parental pair per treatment, i.e., 100 beetles per treatment in total). The field-collected ladybirds were exposed only to the extreme temperature regimes (warm and cold) due to the limited number of individuals available. Field-collected ladybirds were assigned to particular temperature regime at random. This setting resulted in even distribution of pre-overwintering body masses (see below) across temperature regimes for laboratory-reared ladybirds and for two out of three field-collected populations (Supplementary Material File Table S2). For the third population (Nature 3) slightly higher pre-overwintering body mass was observed in the cold treatment (mean mass = 36.13 mg) than in the warm treatment (mean mass = 32.46 mg). Note that variation between individuals within temperature treatments is very high (cold: 26.1–48.9 mg; warm: 24.2–42.8 mg), allowing us to easily analyse the effects of pre-overwintering mass independently of temperature regime.

Over the course of the overwintering experiment, the beetles were checked at monthly intervals (November 16^th^, December 15^th^, January 14^th^, February 17^th^ and March 17^th^, 2016–when the overwintering experiment was terminated). During all the inspections, the Petri dishes with beetles were moved to outdoor conditions to minimize the unwanted warming of the experimental ladybirds, the survival of each ladybirds was recorded, and the watered cotton wool in each Petri dish was replaced with a new piece. Live diapausing ladybirds were commonly tightly attached to the substrate, whereas dead ladybirds dropped off and laid freely on their dorsum. Dead iladybirds were not removed from the experiment to minimize the small risk of false death records (as described in^13^). After the completion of the overwintering stage (on March 17^th^), the ladybirds in the Petri dishes were transported to a room temperature (22 °C ± 1 °C) setting. The next day, the live weight (post-overwintering mass) of all the surviving ladybirds was collected. In the following days, the survival of the ladybirds was checked daily to measure their post-winter starvation resistance (water was provided ad libitum).

### Statistical analyses

Two datasets were created prior to the data analysis. The first dataset (“laboratory”) consisted of the laboratory-reared ladybirds, and this dataset allowed us to investigate the effects of all three winter temperature regimes (warm, normal and cold) as well as to account for the genetic background of the investigated ladybirds (the parents of the investigated ladybirds were known, and their identity could be incorporated as a random effect into the analyses). The raw data serving as the input for the respective analyses are available in the Supplementary Material File (Table S3). The second dataset (“both”) consisted of all the field-collected ladybirds and a subset of the laboratory-reared ladybirds that were exposed to the extreme (warm and cold) winter temperature regimes. This dataset allowed us to compare the effects of the extreme temperature regimes between the laboratory-reared ladybirds and field-collected ladybirds. The raw data serving as input for the respective analyses are available in the Supplementary Material File (Table S4).

To investigate the winter survival of the laboratory-reared ladybirds (employing the “laboratory” dataset) under different winter temperature regimes, a mixed-effects Cox model was run using the “coxme” function implemented in the “coxme” package^58^ in R^59^. Winter survival in months (one to five months) was used as the response variable, parental pair identity was used as a random effect and winter temperature regime (warm, normal or cold), sex (male or female), pre-overwintering mass and all possible interactions between temperature regime, sex and pre-overwintering mass were used as the independent variables in the model. The significance of the differences between particular temperature regimes was tested by Tukey’s HSD post hoc tests using the “glht” function as implemented in the “multcomp” package^60^.

To investigate the effects of the extreme winter temperature regimes on the survival of both the laboratory-reared and field-collected ladybirds (employing the “both” dataset), a Cox proportional-hazards model (Cox-PH) was run using the “coxph” function implemented in the “survival” package^58^ in R^59^. Winter survival in months (one to five months) was used as the response variable, temperature regime (warm or cold), sex (male or female), population identity (Laboratory, Nature 1, Nature 2 and Nature 3), pre-overwintering mass and all the possible interactions between temperature regime, sex, population identity and pre-overwintering mass were used as the independent variables in the model. The survival data were right-censored because some beetles were still alive at the end of the overwintering stage. Tukey’s HSD post hoc tests were employed to reveal the significant differences in the winter survival between beetles originating from the different populations.

To analyse the relative body mass loss during overwintering in the laboratory-reared ladybirds, a linear mixed-effects model (LME) was run using the “lme” function implemented in the “nlme” package^61^ in R^59^. Relative body mass loss was calculated as the proportion of pre-overwintering mass lost during overwintering, i.e., the difference between the pre-overwintering mass and post-overwintering mass divided by the pre-overwintering mass of a given individual. Parental pair identity was used as a random effect in our analysis. Winter temperature regime (warm, normal or cold), sex (male or female), pre-overwintering mass and all the possible interactions between temperature regime, sex and pre-overwintering mass were used as the independent variables in the model. The significance of the differences between the particular temperature regimes was tested by Tukey’s HSD post hoc tests.

To compare the effects of the extreme winter temperature regimes on the relative body mass loss between the laboratory-reared and field-collected ladybirds, analysis of covariance (ANCOVA) was run using the “lm” function in R^59^. Relative body mass loss was used as the response variable, and temperature regime (warm or cold), sex (male or female), population identity (Laboratory, Nature 1, Nature 2 and Nature 3), pre-overwintering mass and all the possible interactions between temperature regime, sex, population identity and pre-overwintering mass were used as the independent variables in the model. Tukey’s HSD post hoc tests were employed to reveal the significant differences in the relative body mass loss between beetles originating from different populations. Analogous model was fitted also for absolute body mass loss data to check whether the difference between sexes can be explained by the existence of sexual size dimorphism (females are the larger sex in *H. axyridis*^51^).

To investigate the post-winter starvation resistance in the laboratory-reared ladybirds, a generalized linear mixed model (GLMM) was run using the “glmmPQL” function implemented in the “MASS” package^62^ in R^59^. The post-winter starvation resistance (i.e., the longevity of adults under no food conditions) was used as the response variable in the model, and a quasi-Poisson distribution of errors was employed. The parental pair identity was used as a random effect in this analysis. The winter temperature regime (warm, normal or cold), sex (male or female), pre-overwintering mass and all possible interactions between temperature regime, sex and pre-overwintering mass were used as the independent variables in the model. The significance of the differences between the particular temperature regimes was tested by Tukey’s HSD post hoc tests.

To compare the effects of the extreme winter temperature regimes on the post-winter starvation resistance between the laboratory-reared and field-collected ladybirds, a generalized linear model (GLM) with a quasi-Poisson distribution of errors was run using the “glm” function in R^59^. Post-winter starvation resistance was used as the response variable, and temperature regime (warm or cold), sex (male or female), population identity (Laboratory, Nature 1, Nature 2 and Nature 3), pre-overwintering mass and all the possible interactions between temperature regime, sex, population identity and pre-overwintering mass were used as the independent variables in the model. Tukey’s HSD post hoc tests were employed to reveal the significant differences in winter survival between beetles originating from different populations.

## Supplementary information

Supplementary information

Supplementary information2

Supplementary information3

Supplementary information4

## Data Availability

All the data produced during the study and analysed in this article are attached as Supplementary Material Files (Tables S1, S3 and S4).
