# Supplementary material for: Effects of the winter temperature regime on survival, body mass loss and post-winter starvation resistance in laboratory-reared and field-collected ladybirds
Source: Sci Rep. 2020 Mar 18;10:4970. doi: 10.1038/s41598-020-61820-7 (PMC7080747; doi:10.1038/s41598-020-61820-7)
Supplement: Supplementary file 1 — Supplementary information [file 41598_2020_61820_MOESM1_ESM.pdf]

Scientific Reports

Effects of the winter temperature regime on survival, body mass loss and post-winter starvation resistance in laboratory-reared and field-collected ladybirds

Michal KNAPP[1*], Michal ŘEŘICHA[1]

[1] Department of Ecology, Faculty of Environmental Sciences, Czech University of Life Sciences Prague, Kamýcká 129, Prague - Suchdol, 165 00, Czech Republic; knapp@fzp.czu.cz, rericham@fzp.czu.cz

* Correspondence:
Michal Knapp, Department of Ecology, Faculty of Environmental Sciences, Czech University of Life Sciences Prague, Kamýcká 129, Praha – Suchdol, 165 00, Czech Republic. Tel. +420 22438 3853; e-mail: knapp@fzp.czu.cz
ORCID: 0000-0003-4487-7317

Supplementary material Figures S1, S2 and Table S2

Figure S1 – Sex specific effects of pre-overwintering body mass on winter survival in *Harmonia axyridis* (Coleoptera: Coccinellidae).

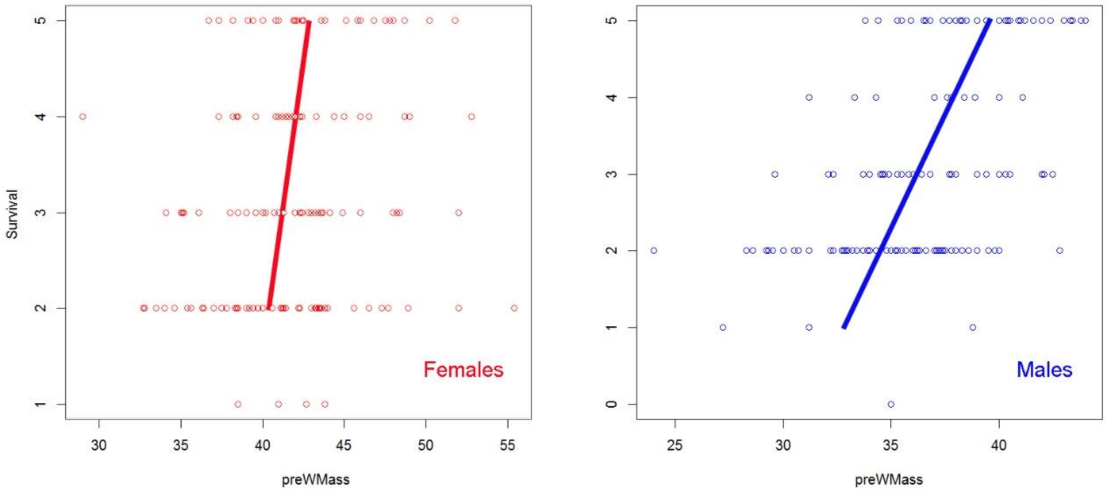

Figure S2 – Differences in pre-overwintering body mass between laboratory-reared (Lab) and field-collected (Nat, Nat2, Nat3) beetles.

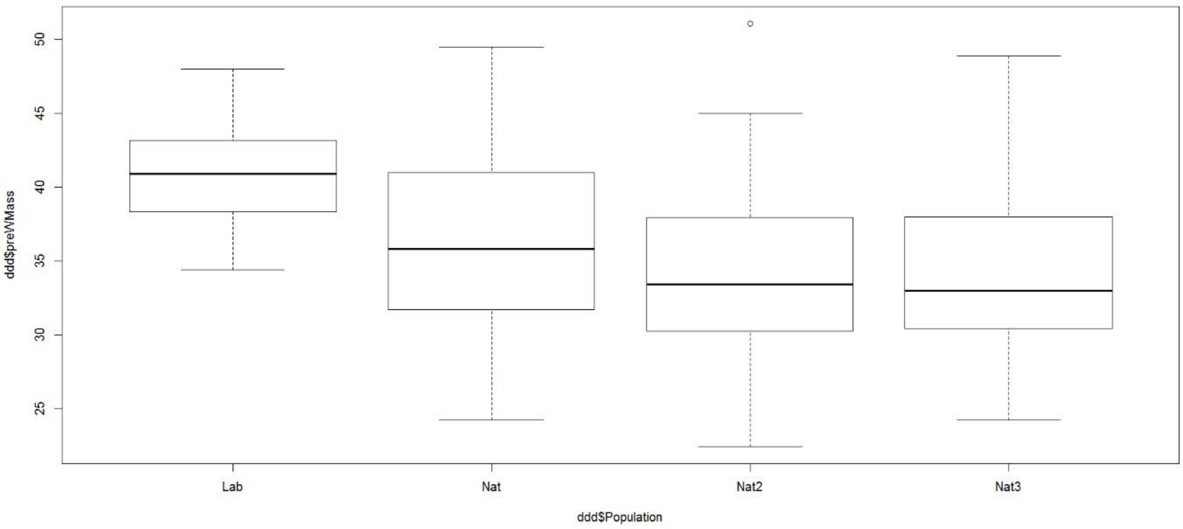

Table S2

Differences in pre-overwintering body mass between temperature treatments within particular populations. Significance of differences was tested using F-tests applied to generalized linear models (GLM) with a Gamma distribution of errors. Separate models were fitted for each population (Lab, Nat1, Nat2 and Nat3).

Population	Df	Df residual	F-value	P-value
**Lab**	2	295	0.747	0.475
**Nat1**	1	50	1.502	0.226
**Nat2**	1	38	0.084	0.774
**Nat3**	1	37	4.620	**0.038**
